# Development of a Robust Sensor Calibration for a Commercially Available Rising Platemeter to Estimate Herbage Mass on Temperate Seminatural Pastures

**DOI:** 10.3390/s24072326

**Published:** 2024-04-05

**Authors:** Jessica Werner, Khaterine Salazar-Cubillas, Sari Perdana-Decker, Kilian Obermeyer, Elizabeth Velasco, Leonie Hart, Uta Dickhoefer

**Affiliations:** 1 Animal Nutrition and Rangeland Management in the Tropics and Subtropics, Institute of Agricultural Sciences in the Tropics, University of Hohenheim, Fruwirthstr. 31, 70599 Stuttgart, Germany; jessica.werner@uni-hohenheim.de (J.W.); sari.perdana@uni-hohenheim.de (S.P.-D.); elizabeth.velasco@lazbw.bwl.de (E.V.);; 2 Institute of Animal Nutrition and Physiology, Kiel University, Hermann-Rodewald-Str. 9, 24118 Kiel, Germany; salazar-cubillas@aninut.uni-kiel.de; 3 Agricultural Centre for Cattle Production, Grassland Management, Dairy Food, Wildlife and Fisheries Baden-Wuerttemberg (LAZBW), Atzenberger Weg 99, 88326 Aulendorf, Germany; kilian.obermeyer@lazbw.bwl.de; 4 Competitiveness and System Evaluation, Agroscope, Tänikon 1, 8356 Ettenhausen, Switzerland

**Keywords:** pasture management, decision support, validation, temperate grasslands, sensor technology

## Abstract

Rising platemeters are commonly used in Ireland and New Zealand for managing intensive pastures. To assess the applicability of a commercial rising platemeter operating with a microsonic sensor to estimate herbage mass with its own equation, the objectives were (i) to validate the original equation; (ii) to identify possible factors hampering its accuracy and precision; and (iii) to develop a new equation for heterogeneous swards. A comprehensive dataset (*n* = 1511) was compiled on the pastures of dairy farms. Compressed sward heights were measured by the rising platemeter. Herbage mass was harvested to determine reference herbage availability. The adequacy of estimating herbage mass was assessed using root mean squared error (RMSE) and mean bias. As the adequacy of the original equation was low, a new equation was developed using multiple regression models. The mean bias and the RMSE for the new equation were overall low with 201 kg dry matter/ha and 34.6%, but it tended to overestimate herbage availability at herbage mass < 500 kg dry matter/ha and underestimate it at >2500 kg dry matter/ha. Still, the newly developed equation for the microsonic sensor-based rising platemeter allows for accurate and precise estimation of available herbage mass on pastures.

## 1. Introduction

Grasslands are an important source of forage for domestic ruminants in Central Europe [1]. Hence, in the European Union, 34% of agricultural land is grassland. In countries like Ireland, the proportions are even greater, with 80–100% of agricultural land [2]. Approximately 31% of dairy cattle, 18% of beef cattle, and even more sheep and goats are kept in systems with access to pastures for at least some time of the year [3].

Seminatural grasslands are ecosystems that are defined as mown and/or grazed permanent grasslands that are not substantially modified by agricultural practices [4]. Despite minimal modification, these ecosystems still require some human intervention, such as reseeding, (organic) fertilization, and biomass removal via livestock or mowing to prevent woody encroachment [3]. Seminatural grasslands are, for example, found in mountainous regions of Central and South Germany, Austria, and Switzerland and are commonly used for livestock grazing [4]. As opposed to temporary, cultivated pastures on arable land, they are characterized by greater species diversity, higher abundancies of herbs, partly lower plant biomass yields, more variable topographic and edaphic conditions, and thus greater spatial heterogeneity [5,6].

Knowledge of available herbage mass is decisive in grazing management to assure sufficient nutrient and energy intakes for grazing animals, to adjust their supplemental feeding in barns with concentrate or additional roughage, and to efficiently use available forage resources on grasslands while avoiding their long-term degradation due to under- or overexploitation [7]. Stocking density as well as the duration and frequency of grazing need to be closely adjusted to the available herbage mass on pastures, particularly in the case of grazing schemes such as strip grazing, rotational grazing, or short-grass grazing (German: Kurzrasenweide) [8,9]. More so, increasing variability in weather conditions and thus plant growth rates between and within years due to climate change may require more opportunistic grassland use [10]. This temporal variation in available herbage mass on pastures may differ depending on other environmental, vegetation, and management factors [11,12,13]. Thus, accurate and precise estimates and regular monitoring of herbage mass on pastures will be even more essential for sustainable and resilient forage use on grasslands in the future.

There is a range of methods to estimate and monitor nondestructive herbage mass on pastures, such as ultrasonic sensors, remote imagery analysis, and rising platemeters, differing in their costs, required infrastructure, adequacy (i.e., accuracy and precision), user-friendliness, and spatial resolution [14]. Remote sensing, for instance, via unmanned-aerial-vehicles (UAV) or satellites, has been suggested as a rapid and automated option for estimating herbage mass [15]. While satellite-based measurements might not offer a sufficiently high resolution for small-scale, fragmented grassland areas, UAV-based estimates may represent a feasible approach for farms employing seminatural grasslands for grazing. However, the cost of UAVs and the required expert knowledge for their application still present obstacles to their wider adoption in small-scale dairy farming [16]. Instead, rising platemeters are commonly used in countries like New Zealand and Ireland in intensive pasture-based dairy systems due to their simplicity and flexibility [17]. Their principle is to estimate herbage mass from compressed sward height (CSH) using (linear) algorithms that were developed from reference datasets based on destructive sampling. Besides vegetation height, CSH might be influenced by canopy ground cover, plant structure, and plant flexibility, which are in turn influenced by seasonal variation in the botanical composition, maturity stage, and nutrient concentrations of the vegetation [18,19]. Those differences in sward characteristics are often a result of different grazing intensities and management strategies, sometimes with highly variable sward patterns in time and space [20,21]. This can further hamper successful herbage mass estimation. Rising platemeters have been successfully calibrated for homogenous, cultivated grasslands dominated by a few plant species, such as perennial ryegrass and clover [22,23,24,25]. However, there is a lack of valid equations and knowledge on decisive factors for an accurate and precise estimation of available herbage mass on temperate seminatural, multispecies grasslands, such as in Central Europe.

It is hypothesized that the existing equation of a commercially available rising platemeter, which was developed in Ireland, is not valid for predicting the available herbage mass on temperate seminatural, multispecies pastures in Central Europe, because of their greater proportions of herb species, variable ground cover, and lower grazing intensities as compared to temporary, cultivated pastures. Hence, it is expected that integrating these factors into prediction equations will allow for a more accurate and precise estimation of the available herbage mass on seminatural, multispecies pastures.

The objectives of the present study were therefore (i) to validate the existing original equation of one microsonic commercial rising platemeter to estimate available herbage mass on temperate seminatural, multispecies grasslands; (ii) to identify vegetation, management, and environmental factors possibly hampering its accuracy and precision; and (iii) if necessary, develop a new equation for botanically and spatially heterogeneous swards on seminatural, multispecies pastures in Central Europe.

## 2. Materials and Methods

A comprehensive dataset was compiled from seven individual research trials to validate the original equation and possibly develop a new equation to predict the available herbage mass on temperate seminatural, multispecies pastures by a commercial rising platemeter. The individual trials were conducted on commercial dairy farms in Eastern Switzerland and Southwest Germany during the vegetation periods of either 2019, 2020, or 2021. The altitude of the farms ranged from 302 to 1297 m above sea level, and annual precipitations and mean annual ambient air temperatures from 665 to 1878 mm and from 6.9 to 11.5 °C, respectively (Table 1). Per trial, one to seven farms were included with each one to nine individual paddocks. All paddocks were grazed by dairy cows or their youngstock in either continuous, rotational, or short grass grazing systems.

### 2.1. Rising Platemeter

In all trials, the CSH was measured with a commercial, semiautomated rising platemeter (Grasshopper Version G2; True North Technology, Shannon, Ireland). The rising platemeter consists of a microsonic sensor, which emits and receives sound waves, placed on the shaft, and a metal plate with an area of 989.8 cm^2^, which falls freely onto the vegetation [26]. The microsonic sensor records the time difference between the transmission of a signal and its reflective return from the plate. The higher the upward displacement of the metal plate, the shorter the time between transmission and return of the reflected signal [27]. The recorded time is then translated into a distance measurement given as mm. The second output of the system is the predicted available herbage mass (kg dry matter (DM)/ha). Data is transferred via Bluetooth to a smartphone or tablet device. More details regarding the sensor system can be found in [26,27]. Available herbage mass is estimated from measured CSH (mm), the targeted residual sward height (cm), and an assumed DM concentration of herbage (g/kg fresh matter). The internal equation (further referred to as the original equation) has been developed for homogeneous, cultivated pastures with perennial ryegrass.

### 2.2. Sampling of the Pasture Herbage

The reference dataset used to calibrate and validate the new equation for the rising platemeter consisted of destructive herbage cuts for all individual trials. The sampled pastures were low-intensity seminatural, permanent grassland with a heterogeneous botanical composition, and some of the paddocks were managed very extensively, because they underlie the directive of protected Natura 2000 habitats [28]. The sampling protocol was similar, but depending on the trial, it differed in the number of measurement points or the size of the sampling area. In Table 1, all the details of the data collection per individual trial are summarized.

For herbage sampling, frames of 0.25 m^2^ (Trials 2, 3, and 4) or 1.00 m^2^ (Trials 1, 5, 6, and 7), which represent the sampling area, were placed randomly within a representative part of the pastures, avoiding dung patches. Within Trials 1, 5, 6, and 7, those frames were also placed within pasture exclusion cages. Those cages were built to simulate the grass regrowth on pasture, preventing cattle from grazing. The vegetation inside and outside of the cages was repeatedly sampled every 2–12 weeks over the grazing season, depending on the trial.

Before cutting, the canopy cover (%) of the herbaceous vegetation within the sampling areas was estimated visually in 5 steps. Moreover, its botanical composition was visually assessed based on the proportion of grasses in total herbaceous biomass according to the classification of the Deutsche Landwirtschafts–Gesellschaft (DLG) [29]: vegetation with >70% of total herbaceous biomass being grasses as grass-rich, with 50–70% of total herbaceous biomass being grasses as balanced, and with <50% of total herbaceous biomass being grasses as rich in clover and other herbs.

The CSH before cutting (CSHpre) was measured by the rising platemeter at 3–5 measurement points per sampling area (Table 1). Using either manual or automated grazing scissors (Makita DUM604; Anjō, Japan), depending on the individual trial, the above-ground herbage mass was then cut at 35–40 mm above ground level. Similar to CSHpre, the CSH immediately after cutting (CSHpost) was quantified at 3–5 points per sampling area. Mean CSHpre and CSHpost were taken across the respective 3–5 measurements per sampling area. The CSH of the harvested herbage mass was calculated by subtracting CSHpost from CSHpre and used to estimate the available herbage mass using the original equation of the rising platemeter.

The reference value for the available herbage mass (kg DM/ha) was calculated based on the pasture cuts. The harvested fresh biomass per sampling area was weighed. The DM concentration (g/100 g fresh matter) was determined with different methodologies per trial (see Table 1). It was then used to calculate the reference herbage mass (kg DM/ha) and, subsequently, herbage density (kg DM/m^3^) from the CSH of harvested herbage (=herbage mass (kg DM/ha)/(CSH (cm) × 100)).

Climate data for trials 1, 5, 6, and 7 were mainly recorded on the farm using a HOBO^®^ U23 Pro External Temperature/Relative Humidity Data Logger for measuring the ambient air temperature. Precipitation was recorded by a HOBO^®^ Rain Gauge (Metric) Data Logger RG3-M (Onset Computer Corporation, Bourne, MA, USA). Missing data, especially annual values, were supplemented similarly to all climate data in Trials 2, 3, and 4 by values derived from close-by weather stations (WetterKontor GmbH, Ingelheim am Rhein, Germany).

Descriptive parameters such as season, slope, and grazing system were defined manually, whereby spring was defined as April–May, summer as June–July, and autumn as September–November. The slope was visually assessed by the sampling person based on the topography of the whole paddock and categorized as flat, hilly, or steep. The categories of the prevalent grazing system per farm were defined as continuous grazing if there was no change of paddocks, and the animals had access to a greater area of pasture over a long period, whereas rotational grazing was defined as changing paddocks daily or after a few days. Short-grass grazing is a continuous grazing system in which the sward height is maintained between 3 and 5 cm, which is much shorter than in conventional continuous grazing systems at approximately 8 cm [11,30].

### 2.3. Statistical Analysis

All statistical analyses were conducted using R statistical software version 4.2.3 (R Core Team, Vienna, Austria) and Microsoft Excel (Microsoft Inc., Seattle, WA, USA; Version 2019).

A total of 2114 observations were used to evaluate and improve the prediction of available herbage mass. Observations with incomplete data, where at least one independent variable was missing, were excluded from the analysis, as were those with a DM concentration of >50 g/100 g fresh matter, a canopy cover of <70%, a negative CSH, or a herbage density of <5 g DM/m^3^. A Grubb outlier test [31] was performed thereafter to remove the statistical outliers. As a result, the final dataset, further referred to as dataset 1, was used to evaluate and improve predictions of available herbage mass and consisted of 1511 observations after removing n = 603 (28.5%) of individual observations that were considered illogical values or outliers. Those values were not equally distributed across trials (Table 1).

For each observation, there were data on trial ID, farm ID, pasture ID, sampling position (i.e., inside the exclusion cage (in) or outside (out)), date, season, altitude, mean annual ambient air temperature, mean annual precipitation, as well as mean ambient air temperature and mean precipitation two weeks prior to the sampling event. Further, there was information on the prevalent grazing system, as well as CSHpre and CSHpost, canopy cover, botanical composition, DM concentration, and density of the herbage mass. The reference value per observation was the available above-ground herbage mass (i.e., observed herbage mass), determined by destructive harvest (Table 2).

In the first step, the original equation was evaluated using Dataset 1 by comparing observed herbage mass to predicted herbage mass by using default values with a constant DM concentration of herbage of 16 g/100 g fresh matter and a residual sward height of 4 cm (Equation (1)). As mentioned in Hart et al. [32], there is an option to change the default settings of the equation in the smartphone application of the Grasshopper. In the present study, it was, therefore, further investigated to see if herbage mass predictions improve when the default value of 16 g DM/100 g fresh matter is substituted by the measured value of the laboratory analysis, further referred to as Equation (2). The residual sward height remained 4 cm in both cases.

It was also analyzed to see if mean values of available herbage mass per paddock might be more feasible to predict by the rising platemeter to reduce the effect of spatially heterogenous biomass distribution. For this, Dataset 1 was grouped by trial, farm, pasture, in- or outside the exclusion cage, and sampling event to form Dataset 2, with *n* = 360 observations. The mean of the observations was calculated for the quantitative variables, whereas the mode was calculated for the qualitative variables.

For the improvement of the prediction of available herbage mass by the rising platemeter, two approaches were tested and evaluated. Nonlinear regression and linear regression. The nonlinear regression entailed adjusting the coefficients of the original equation to Dataset 1, while maintaining the same independent variables and their relationships. The multiple linear regression approach was applied using a forward and backward stepwise process with observed herbage mass as a dependent variable and inside or outside of exclusion cages, season, CSHpre, CSHpost, canopy cover, botanical composition, DM concentration of the harvested herbage mass, altitude, temperature, precipitation, and grazing system as independent variables.

A leave-one-out cross-validation was used to select the appropriate approach for developing a new equation that predicts herbage mass. For this, the total of 1511 individual observations were divided randomly into five subsets consisting of four training sets and one test set. Then, the root mean squared error (RMSE) of the four equation options developed with the training datasets was calculated for the test dataset. The same procedure was repeated five times, with different test sets each time. The model structure with the lowest RMSE was chosen for developing the new equation that predicts available herbage mass. The RMSE for the nonlinear regression ranged from 535 to 541 kg DM/ha, whereas the RMSE for the linear regression was lower at 428 kg DM/ha. Both nonlinear and linear regressions exhibited a coefficient of variation of 6% for the RMSE calculated across the five datasets. Therefore, the new equation, further referred to as Equation (3), was modeled using multiple linear regression.

For this, all observations (*n* = 1511) were used to identify the coefficients and independent variables of the new equation (Equation (3)). Multiple linear regression was performed with the same independent variables as previously described, including quadratic and cubic terms, as well as two-level interactions. The RMSE, mean absolute error, coefficient of determination, variance inflation factor, Akaike information criterion, and Bayesian information criterion were determined for each developed equation. To evaluate the accuracy and precision, predictions with the lowest RMSE, mean absolute error, Akaike information criterion, Bayesian information criterion, greater coefficient of determination, and a variance inflation factor of <10 were considered the most accurate and precise [33].

Statistical parameters were calculated for all the equations used. The concordance correlation coefficient (CCC) and RMSE were calculated to measure the precision and adequacy of the predictions of the original equation using measured and fixed independent variables. As a combined measure of accuracy and precision, the CCC ranges from −1 to 1, with a value of 1 denoting perfect agreement between observed and predicted herbage mass [34,35]. The CCC was partitioned into a correlation coefficient that measures precision and a bias correction factor coefficient that measures accuracy [36]. The RMSE measures the adequacy of the predictions and is calculated as the square root of the mean squared prediction error (MSPE) between the observed and predicted herbage mass (expressed as kg DM/ha) and the percentage of the mean observed herbage mass. The MSPE was further partitioned into error due to central tendency (i.e., overall bias), error due to regression, and error due to disturbance (i.e., random error) [36].

Residuals between observed and predicted herbage mass with the original and new equations were calculated and expressed as absolute (kg DM/ha; observed − predicted) and relative values (% mean; absolute residual × 100/observed mean). Additionally, absolute and relative residuals were expressed as the actual magnitude, regardless of their sign. The residuals were then classified by dependent and independent variables to identify characteristics that are associated with large residuals. A pivot table in Microsoft Excel (Microsoft Inc., Seattle, WA, USA; Version 2019) was used to categorize residuals according to qualitative independent variables.

To assess the goodness of fit of the regression model, a residual plot comparing residual available herbage mass (*x*-axis) and predicted herbage mass (*y*-axis) was built (Figure S1; Supplementary Materials). Further, a scatter plot was created in Microsoft Excel plotting the observed herbage mass against the relative absolute residual (i.e., relative residuals regardless of their sign) in the % of observed herbage mass, including the mean bias of observed and predicted herbage mass and the 90% confidence intervals for the observed biomass categories < 500 kg DM/ha, 500–1000 kg DM/ha, 1000–1500 kg DM/ha, 1500–2000 kg DM/ha, 2000–2500 kg, and >2500 kg DM/ha.

## 3. Results

The observed herbage mass ranged from 59 to 3538 kg DM/ha. All observations were measured during April–November in the respective years, with most measurements obtained in summer (June–August; 52%) and less in spring (18%) and autumn (30%). The environmental conditions and vegetation largely varied across the different sampling areas. For instance, the precipitation varied from 0.4 to 285.4 mm two weeks prior to the sampling event, as did the mean daily ambient air temperature, which ranged from 3.5 to 20.6 °C two weeks before the sampling event. Additionally, the botanical composition of the herbaceous vegetation was very heterogeneous, with 49% of all observations being conducted on balanced swards, 18% on grass-dominated swards, and 33% on herb-dominated swards. There was additionally a great variation in the topography of the individual sampling area, with 24% of the samplings being conducted on steep and 43% on hilly paddocks.

### 3.1. Evaluation of the Original Equation Using Measured or Constant Dry Matter Concentration

**Figure 1:**
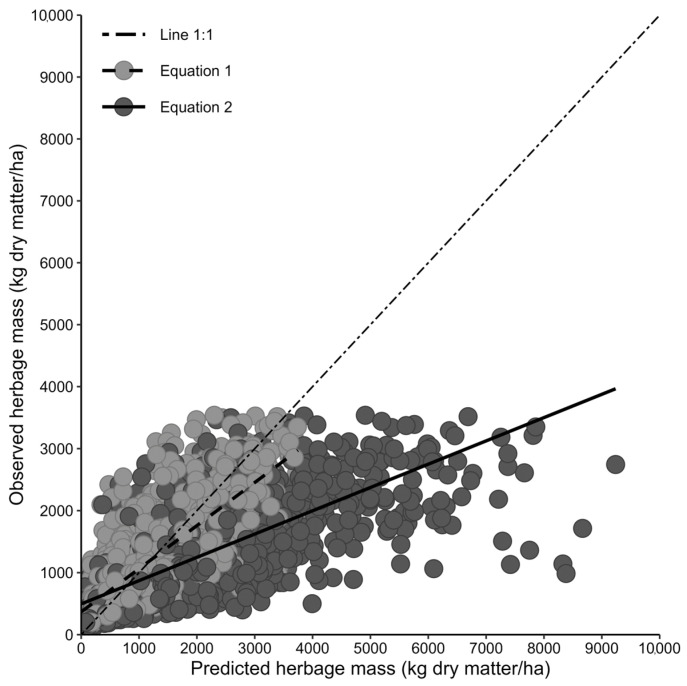
Relationships between observed and predicted herbage mass on temperate seminatural, multispecies pastures when predicted with the original Equations (1) and (2) ^1^ (dataset 1; *n* = 1511 observations). ^1^ Equation (1) considers fixed values for herbage dry matter concentrations (i.e., 16 g/100 g fresh matter) and residual sward height (i.e., 4 cm); Equation (2) considers the measured herbage dry matter concentration and a fixed residual sward height (i.e., 4 cm).

The first step of the present study was to evaluate the performance of the existing original equation to predict the available herbage mass. For this, the above-ground herbage mass was estimated with the original equation either by using a constant DM concentration of 16 g/100 g fresh matter (Equation (1)) or the actually measured DM concentrations of each individual herbage sample (Equation (2)). The RMSE of the predicted herbage mass was 540 kg DM/ha (44.3% of the observed mean) and 1291 kg DM/ha (106% of the observed mean) when a constant or the measured DM concentration were used, respectively (Table 3, Figure 1). The partitioning of the CCC indicated that the original equation (Equation (1)) predicted herbage mass with high accuracy (bias correction factor coefficient = 0.99) but lower precision (correlation coefficient = 0.80).

**Figure 2:**
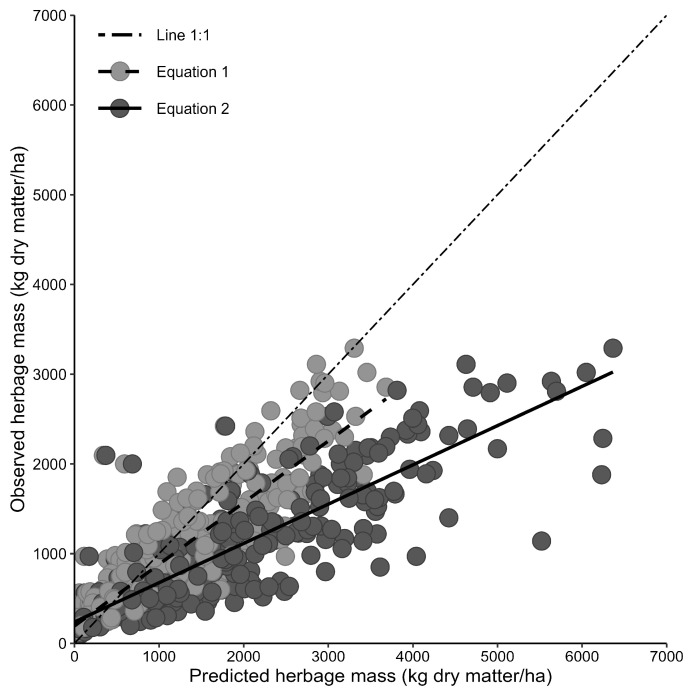
Relationships between observed and predicted herbage mass on temperate seminatural, multispecies pastures when predicted with the original Equations (1) and (2) ^1^ based on the grouped dataset per paddock (dataset 2; *n* = 360 observations). ^1^ Equation (1) considers fixed values for herbage dry matter concentrations (i.e., 16 g/100 g fresh matter) and residual sward height (i.e., 4 cm); Equation (2) considers the measured herbage dry matter concentration and a fixed residual sward height (i.e., 4 cm).

In a second step, the dataset was averaged per paddock (i.e., grouped) to test if the mean herbage mass per pasture (dataset 2; *n* = 360) can be predicted more adequately as compared to that in individual sampling areas (*n* = 1511). The mean observed herbage mass per paddock and, consequently, the RMSE were lower than when individual sampling areas per paddock were considered (Table 4). However, observed herbage mass was still increasingly overestimated (Figure 2), irrespective of whether measured (Equation (2)) or constant DM concentrations (Equation (1)) were used, and relative RMSE was greater when evaluated based on mean compared to individual values.

Residuals of the original equation (Equation (1)) were lowest for grass-rich swards (384 kg DM/ha and 40% of the mean observed herbage mass) in comparison to swards with balanced species composition (408 kg DM/ha and 38% of the mean observed herbage mass) or herb-rich pastures (398 kg DM/ha and 39% of the mean observed herbage mass). However, absolute residuals were lowest for the short grass grazing system (308 kg DM/ha; 44% of mean observed herbage mass), followed by the continuous grazing systems (420 kg DM/ha and 38% of mean observed herbage mass) and the rotational grazing systems (427 kg DM/ha and 37% of mean observed herbage mass).

### 3.2. Development of a New Equation

Evaluation of the original equation revealed that more specific calibrations are needed to predict above-ground herbage mass on temperate seminatural and multispecies pastures. Therefore, two approaches were used to develop a new equation to improve the prediction of herbage mass from the CSH as measured by a rising platemeter: a nonlinear regression and a multiple linear regression approach. The nonlinear regression approach obtained a mean RMSE ranging from 535 to 541 kg DM/ha, whereas the multiple linear regression performed better with a mean RMSE of 428 kg DM/ha.

Therefore, the multiple linear regression approach was chosen for developing a new equation to estimate herbage mass on seminatural, multispecies pastures based on dataset 1 (*n* = 1511). The retained independent variables included CSHpre, the canopy cover of the herbage, and the altitude of the farm, as well as corrections for differences in the pasture slope (i.e., flat, hilly, and steep; Table 5).

Residual plots (Figure S1; Supplementary Materials) showed that there was no clear pattern in the residual distribution; therefore, the developed equation fitted best to the prevalent dataset. In the same line, the absolute residuals of predicted herbage mass by the new equation were similar for short grass grazing systems (242 kg DM/ha, 46% of observed mean biomass), continuous grazing systems (361 kg DM/ha, 39% of observed mean biomass), and rotational systems (324 kg DM/ha, 30% of observed mean biomass).

**Figure 3:**
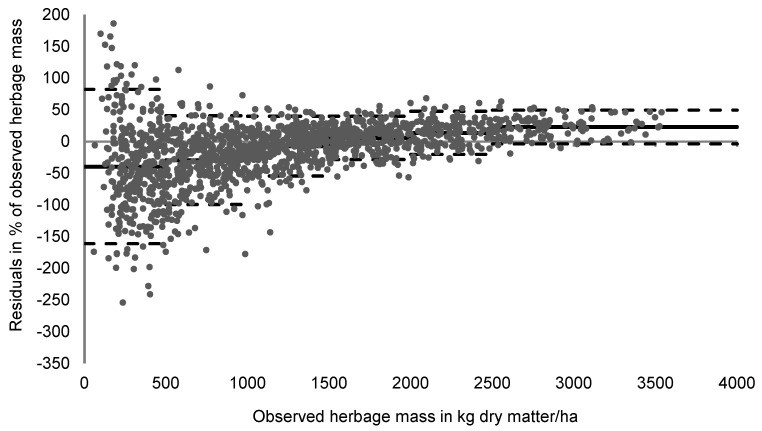
Observed herbage mass plotted against the residuals in % of the observed herbage mass, including the mean bias (solid line) and the 90% confidence interval (dashed lines) per category (<500, 500–1000, 1000–1500, 1500–2000, 2000–2500, and >2500 kg dry matter/ha; *n* = 1511).

In a graphical analysis (Figure 3), different categories of observed biomass were analyzed. At very low herbage mass (<500 kg DM/ha), the new equation appeared to overestimate availability, whereas the latter was underestimated at very high herbage availability (>2500 kg DM/ha). Additionally, it demonstrates that the mean bias is greatest (showing a mean overestimation of 29% of observed biomass) and the 90% confidence interval widest, ranging between 82 and −161%, at an herbage mass < 500 kg DM/ha. The residuals were considerably lower (i.e., the predictions were more accurate) when the observed herbage mass ranged between 1000 and 1500 (mean bias = 7%; confidence interval between −54 and 40%) or between 1500 and 2000 kg DM/ha (mean bias = 6%; confidence interval between −28 and 40%).

## 4. Discussion

The present study aimed at evaluating an existing equation of a rising platemeter on temperate, seminatural, multispecies pastures. A database was created from simultaneous rising platemeter measurements and available herbage mass determined by destructive sampling on a range of farms in southwest Germany and eastern Switzerland. It was hypothesized that the original equation of the commercial rising platemeter is not valid for typical pastures in these regions due to the prevalent multispecies pastures. To test this hypothesis, the results of the observed herbage mass were compared to the estimates derived by the original equation, which was developed in Ireland.

### 4.1. Existing Equation Is Not Valid for Seminatural, Multispecies Pastures

The adequacy of the original equation was low (RMSE of 44.3% of mean observed herbage mass for Equation (1)), which confirms the results of various earlier studies of the poor validity of existing equations of rising platemeters when applied to either multispecies grasslands or more extensively managed pastures and grasslands [37].

One explanation for the limited adequacy of the existing equation in the present study may be differences in the sward characteristics between the calibration or validation datasets. The equation of the Grasshopper rising platemeter was developed on Irish grasslands, which are characterized by homogenous swards with a high abundance of perennial ryegrass, sometimes mixed with red and white clover [38]. Moreover, these pastures are intensively used at high stocking rates and with high fertilization rates [39,40]. The postgrazing sward height is set to 3.5–4 cm, while the pregrazing height should not exceed 16 cm [41,42].

In contrast to this, the validation dataset of the present study was collected on seminatural grasslands. On those grasslands, a high abundance of white and red clover, as well as other herbs, are prevalent and are used less intensively than the pastures in Ireland [3]. The grazing management of these pastures in southwest Germany and eastern Switzerland is quite diverse [43]. Some farms implement rotational schemes, while others continuously graze their pastures with variable or no targets in pre- and postgrazing sward heights. Due to the grazing intensity and the selectivity of grazing animals, a patchy and spatially heterogenous sward structure evolves [44,45], with differences in botanical composition between frequently grazed and ungrazed areas. Further, more extensive grazing leads to a greater proportion of tall and stemmy vegetation [45]. For the rising platemeter, this occurrence of stemmy vegetation has an impact on the resistance to plate pressure and therefore the compression of the herbage mass, which is different from that of short and predominantly vegetative swards [46].

The low adequacy of the original equation in predicting available herbage mass on these seminatural, multispecies pastures was mainly due to reduced precision and not poor accuracy, as indicated by a great share of the total mean squared prediction error that was due to regression (error due to regression = 25%; central tendency = 0.01%) and the CCC coefficients (correlation coefficient = 0.80; bias correction factor coefficient = 0.99). The reduced precision could be due to the great range of grassland types and management systems covered in the present dataset, with a high variation in almost all input variables and available herbage mass. In the same line, spatial heterogeneity was very high in species composition and, with this, sward structure and flexibility, which in turn influenced the relationship between CSH and available herbage mass.

An additional reason for the reduced precision of the original equation could be the fact that steep slopes may hamper accurate estimation of CSH with rising platemeters. Given the fact that the metal plate of the measurement tool needs to be parallel to the ground, tilting of the metal plate on steep slopes may result in greater errors in the CSH measurement. Mean relative residuals in the present study were indeed lower for flat sampling areas (326 kg DM/ha) as compared to those in hilly (441 kg DM/ha) or steep (429 kg DM/ha) locations of the pastures, which may have contributed to a lower precision of predictions of available herbage mass. Finally, variation in actual DM concentrations of herbage in the present study was very high (mean 26 g/100 g fresh matter; one standard deviation 7.8), which would at least partly also explain the low precision of the original equation. Mean DM concentrations in the present study were greater than the constant 16 g/100 g fresh matter used for predictions, resulting in a greater bias (i.e., lower accuracy) and an increasing overprediction of herbage mass with rising herbage mass on pastures.

Adequacy was slightly improved when analyzing the dataset of mean sward measurements and herbage mass per paddock, as indicated by a greater CCC due to an increase in the Pearson correlation coefficient as compared to predictions based on individual observations. Nevertheless, these improvements were not substantial. Moreover, residuals were high across all vegetation types, seasons, slopes, and grazing systems, suggesting that other or a combination of different factors and their interactions are responsible for the lack of precision.

### 4.2. Selected Variables for the New Equation

The evaluation of the original equation revealed that it is not valid for temperate seminatural, multispecies pastures. Therefore, a new equation was developed in two steps. Firstly, by only adjusting the coefficients but keeping the same equation structure as Equation (1) and choice of input variables; and secondly, by developing new equations with additional input variables.

Solely adjusting the coefficients of the input variables in the original equation did not improve its adequacy substantially (RMSE reduced from 540 to 537 kg DM/ha), indicating that further variables need to be included to improve the precision and reduce the random error of predictions of available herbage mass on seminatural, multispecies pastures. Possible additional variables for a new equation were selected, with the aim of only including variables that are easy to measure and using as few variables as possible to decrease the error. The variables selected for the new equation were, besides the CSHpre obtained by the rising platemeter, the slope of the paddock (i.e., flat, hilly, and steep), the canopy cover of the vegetation, and the altitude.

Among the selected predictors, the altitude could be easily derived from a GPS sensor attached to the rising platemeter and thus measured for individual sampling areas. Altitude estimates from GPS data are, however, subject to large error, depending on, for instance, the number of satellites available, their distribution and signal strength, and speed of movement or duration of measurements [47]. However, as compared to the other input variables (see below), predictions of available herbage mass were not very sensitive to uncertainties in altitude. For instance, according to the coefficient estimate, differences in altitude of 100 m above sea level would only result in an increase or decrease in the predicted available herbage mass of 39 kg DM/ha. Hence, an average altitude per paddock could also be calculated or taken from a general farm map without largely compromising the precision of herbage mass predictions.

Altitude may indirectly influence the relationship between CSH and available herbage mass due to, for instance, differences in ambient temperature and precipitation, and consequently, in botanical composition, plant structure, and maturation, and thus the rigidity of the sward. In this line, altitude correlated negatively with the mean annual ambient air temperature (r = −0.64) and positively with annual precipitation (r = 0.40) or herbage DM concentration (r = 0.25; data not shown). Additionally, the pastures at higher altitudes were rather herb-rich or of mixed species composition, whereas the pastures at lower altitudes were dominated by grasses.

Additionally, the slope of the paddock was selected during multiple linear regression analysis, in line with the differences in residuals between paddocks of different slope categories (i.e., flat, hilly, and steep) when the original equation was used (see previous section). Hence, including slope in the equation resulted in lower and similar mean residuals across slope categories (280–330 kg DM/ha). Similar to altitude, this information could possibly be derived from geographic coordinates or the general information available for each paddock from the farm maps developed with the rising platemeter.

The coefficients for the slope factor were similar for hilly (134 kg DM/ha) and steep paddocks (140 kg DM/ha), but greater than four flat paddocks (0 kg DM/ha), suggesting that on sloped pastures, herbage mass would be underestimated by equations without a slope factor. The effect of slope cannot be fully explained; yet, the underestimation may be due to the fact that on slopes, the metal plate of the RPM tilts, increasing its distance to the ultrasonic sensor, and thus resulting in lower estimates of CSH and herbage mass. Moreover, the rigidity of plants may be lower on sloped ground than on flat ground [20]. For instance, there may be greater species diversity observed on steeper slopes, which can be related to a lower risk of invasion by high-growing competitive species than on flatter grounds.

The interaction between slope and CSH was also retained in the newly developed equation. For hilly and steep pastures, biomass estimates per unit of CSHpre were reduced by −3.5 and −4.9 kg DM/ha, respectively, equivalent to 19.7% and 27.7% of the mean available herbage mass per mm of CSHpre (17.7 kg DM/ha). Such differences could again be due to differences in the botanical composition, plant structure, and vegetation density between flat, hilly, or steep pastures. At a mean CSHpre of 83 mm, this would yield considerable differences in the estimated available herbage mass of 288 kg DM/ha on hilly and 403 kg DM/ha on steep pastures as compared to flat pastures.

Lastly, the canopy cover of vegetation on pasture was retained in the newly developed equation. The rising platemeter is expected to capture differences in vegetation density and, hence, indirectly, canopy cover when measuring the CSH, with lower canopy cover leading to lower vegetation density and thus a lower CSH [48]. Nevertheless, there were no correlations between canopy cover or vegetation density and CSHpre in the present study (data not shown), suggesting that plant rigidity and height are more decisive than canopy cover or vegetation density for CSH measurements in seminatural, multispecies pastures.

The inclusion of the canopy cover considerably improved the adequacy of the herbage mass predictions and greatly affected absolute mass estimates. For instance, consideration of canopy cover as a predictor variable decreased RMSE to 430 kg DM/ha as compared to 452 DM/ha when only CSHpre, slope, and their interaction were incorporated into the model (data not shown). Moreover, a canopy cover greater or lower by 10% would increase or decrease herbage mass estimates by 150 kg DM/ha, according to the new equation. Hence, differences in canopy cover should be considered in herbage mass predictions, particularly in seminatural, multispecies pastures with greater variation in canopy cover (from 70 to 100% in the present dataset) as compared to temporary, cultivated pastures for which the original equation had been developed.

Nevertheless, precise estimates of canopy cover are difficult to obtain. They may be determined by camera systems, but they are commonly only estimated visually. These visual estimations must be conducted for each sampling area and are subject to large errors, likely person-specific, and are thus limited in their reproducibility. Hence, a clear, standardized guideline is needed in order to increase the adequacy of canopy cover estimates and, consequently, of herbage mass predictions on seminatural, multispecies pastures.

Other factors such as CSHpost, grazing management system, precipitation, ambient temperature, month, week, season, botanical composition, and flexibility were not included in the model because their inclusion increased the Akaike information criterion and Bayesian information criterion, the R^2^ did not improve substantially, or they were strongly correlated with input variables already retained in the model.

### 4.3. Adequacy of the Newly Developed Equation

The mean RMSE of the predictions using the newly developed equation was 421 kg DM/ha (i.e., 35% of the observed mean). The mean bias across the entire range of available herbage mass was very low (i.e., very high accuracy). Hence, the key limitation of the equation was reduced precision, which may hamper the use of the platemeter in grazing research and management. A literature review by Murphy et al. [9] showed that precision and thus the adequacy of rising platemeter predictions are commonly lower for grasslands with mixed species composition than those dominated by perennial ryegrass (with or without white clover). Despite reducing the adequacy of predictions, the precision of herbage mass predictions in the present study was still high, with an R^2^ of 0.71 and greater than those in previous research focusing on multispecies grasslands. Hence, R^2^ values of herbage mass predictions by rising platemeters were only 0.31, 0.35 to 0.83, and 0.63 in studies by Sanderson et al. [49], Martin et al. [19], and Litherland et al. [50] for mixed species grasslands in the USA, Canada, or New Zealand, respectively. Instead, adequacy and precision were similar or only slightly poorer than those reported for homogenous perennial ryegrass grasslands in previous studies testing platemeters or other indirect sensor systems. For instance, Klootwijk et al. [23] determined that predictions of available herbage mass on intensively grazed perennial ryegrass pastures in the Netherlands by a rising platemeter had a RMSE of 25–31% of the observed herbage mass with an R^2^ value of 0.83. A similar study in Ireland on trial plots and grazed paddocks dominated by perennial ryegrass found that a rising platemeter predicted herbage mass with an error of 354 kg DM/ha and a residual prediction error of 27.1% [9].

Relative residuals in the present study were similar across different vegetation types, seasons, and slope categories, but were slightly greater in pastures dominated by grasses, and for continuous and short grass grazing systems due to their relatively low herbage mass. Hence, as per Figure 3, there was a tendency to underestimate herbage mass at very high herbage availability, and overestimate it at very low above-ground herbage mass. Accordingly, mean bias exceeded ±25% at an observed herbage mass of <500 kg DM but was lower than ±15% of the observed mean at an available herbage mass of 1000–2500 kg DM/ha. Hence, the adequacy of predictions appears to be greater at intermediate herbage availability, irrespective of the season, slope, and vegetation type, whereas the use of the newly developed equation for pastures with very low herbage availabilities, such as pastures used in short grass grazing systems, on poor soils, or during dry spells, may be associated with greater levels of uncertainty.

Looking at the presented results as well as the literature, it might not be possible to obtain highly accurate, precise, and robust equations for predicting herbage mass on seminatural, multispecies grasslands with low sward heights from rising platemeter measurements. On the one hand, there are limitations related to the reference method of destructive harvest of available biomass itself. For instance, some errors in the present reference dataset may be due to the fact that destructive sampling was conducted at different cutting heights, by different people, and either by manual or electric shears [51]. Additionally, some cut plant material might not have been collected (e.g., small particles falling on the ground). On the other hand, CSH measurements are subject to errors. Usually grass-dominated vegetation on grasslands with very low sward heights has a great proportion of stem material and few leaves, especially on grazed pastures [52]. The stems are more resistant to compression than the leaves, thus resulting in an overestimation of available herbage mass based on CSH measurements. Similar technical limitations may explain the underestimation of the available herbage mass > 2500 kg DM/ha because the CSH measurement of the rising platemeter is limited to sward heights below 22.3 cm [46] and has only been validated up to a CSH of 17.8 cm [27].

At the same time, it is difficult to define which level of accuracy and precision is acceptable, because this will vary depending on the purpose for which the predictions are used (e.g., grassland monitoring, grazing management, farm documentation, and research). According to Sanderson et al. [49], an error level of <10% is necessary from an economic point of view to reimburse the investments in labor and tools. However, it might not be possible to reach such high levels of adequacy in seminatural, multispecies grasslands, in particular not when herbage mass is very low, because (inevitable) measurement and estimation errors are quite high when expressed relative to actual herbage mass on pastures. Moreover, the present dataset was very versatile, with a mean observed herbage mass of 1218 kg DM/ha, but ranging from 59 to 3538 kg DM/ha, which also challenges the development of an equation that is valid across the entire range of herbage availabilities.

Further, it may be very difficult to reach those aimed levels of accuracy by visual estimation if this is set to be the standard procedure on the farm. Especially at low levels of available herbage mass. For instance, at a herbage mass of 500 or 2500 kg DM/ha, 10% would equal to only 50 or 250 kg DM/ha.

Considering that several samples need to be taken to obtain an accurate estimate of herbage mass by destructive sampling [19], and that farmers only have limited time and resources to conduct such measurements, an easy-to-apply, robust method is still needed that offers more accurate results than visual estimations.

Nevertheless, new methods of data analysis such as machine learning in combination with the use of additional easily accessible predictor variables (e.g., weather variables) should be explored in order to improve the precision of the predictions of herbage mass and regrowth rates in seminatural, multispecies grassland used for livestock grazing [24,53]. Finally, the precision of herbage mass estimates may be at least partly compensated by increasing the number of measurements per paddock and developing detailed measurement protocols for seminatural, multispecies pastures. Combined with robust and reliable sensor technologies, this may help to reduce the hesitance to implement more digital solutions for grazing management on commercial farms.

## 5. Conclusions

The original equation of the tested commercial rising platemeter developed to estimate the available herbage mass from CSH on homogenous, intensively grazed pastures was not adequate for temperate seminatural, multispecies pastures. The new equation developed in the present study includes, besides CSH, altitude of the farm (m above sea level), canopy cover of the herbaceous vegetation (%), and slope of the paddock as additional variables. The adequacy of the new equation (RMSE of 421 kg DM/ha) is greater than that of the original equation (RMSE of 540 kg DM/ha), in particular at intermediate herbage mass on pastures, and is still high given the highly heterogenous vegetation on the studied pastures. However, the new equation may overestimate herbage mass at a very low herbage mass (<500 kg DM/ha). Shortcomings in the precision of predictions (R^2^ = 0.71) may be compensated by increasing the number of measurements per paddock and by establishing a standardized protocol. Together with the development of reliable and robust sensor technologies, the improved precision and adequacy would help to implement digital solutions to grazing management to contribute to a more sustainable and resilient forage use.

## Figures and Tables

**Table 1:** Origin and conditions of data collection used to validate an original equation and develop a new equation of a commercial microsonic-based rising platemeter to estimate herbage mass on seminatural, multispecies pastures in Central Europe (arithmetic mean ± one standard deviation (when available)).

Trial	Year	Farms (*n*)	Altitude (m above Sea Level)	Annual Precipitation ^1^ (mm)	Annual Ambient Air Temperature ^1^ (°C)	Number of Paddocks (*n*)	Paddock Size (ha)	Sampling Area (m^2^)	Measurement Points er Sampling (*n*)	Observations per Trial before Cleaning (*n*)	Observations per Trial after Cleaning (*n*)	Sample Drying
1	2019	7	580 ± 202	975 ± 104	9.9 ± 1.2	4 ± 2.8	3.0 ± 1.7	1.00	5	287	220	45 °C 72 h
2	2019	1	682	1212	8.4	1	3.0	0.25	3	283	264	100 °C 24 h
3	2019	2	1087 ± 202	1174	7	5 ± 0.5	9.3 ± 2.5	0.25	5	533	482	60 °C 48 h
4	2019	1	534	1878	10.5	1	-	0.25	3	39	37	60 °C 48 h
5	2020	7	536 ± 206	774 ± 79	10.1 ± 1.1	3 ± 2.8	4.0 ± 1.3	1.00	5	429	290	45 °C 72 h
6	2020	4	916 ± 80	1143	8.4	3 ± 0.5	7.8 ± 2.1	1.00	5	305	113	45 °C 72 h
7	2021	4	919 ± 72	1324	6.9	2 ± 0.7	8.9 ± 4.0	1.00	5	238	105	45 °C 72 h

^1^ Annual weather data were derived from weather stations close to each farm (WetterKontor GmbH, Ingelheim am Rhein, Germany) and were averaged per trial.

**Table 2:** Descriptive statistics of the dependent and independent variables related to agroecological conditions, management, and available herbage mass on temperate seminatural, multispecies pastures in Central Europe (*n* = 1511).

Dependent Variable		Mean	Standard Deviation	Min	Median	Max
Herbage mass	kg dry matter/ha	1218	774	59	1088	3538
**Independent variables**						
Month	from 0 to 12	7	2	4	7	11
Week	from 0 to 52	30	8	17	29	45
Altitude	m above sea level	799	284	302	807	1297
Precipitation ^1^	mm	88.6	46.9	0.4	77.3	285.4
Annual precipitation	mm	1100	219	665	1174	1878
Mean ambient air temperature ^1^	°C	13.6	4.5	3.5	15.2	20.6
Annual temperature	°C	8.7	1.3	6.9	8.4	11.5
Canopy cover	%	91	8	70	90	100
CSHpre	mm	83.2	39.0	4.2	79.0	215.4
CSHpost	mm	40.5	16.2	0.2	38.6	103.8
CSHpre-CSHpost	mm	42.7	29.7	0.8	34.8	163.2
Dry matter	g/100 g fresh matter	26.0	7.8	7.0	24.0	50.0
Density	kg ha/m^3^	141	61	50	134	465
Season	spring (18%), summer (52%), autumn (30%)					
Sampling position	inside the cage (16%), outside the cage (84%)					
Botanical composition	grass-rich (18%), herb-rich (33%), balanced (49%)					
Slope	flat (33%), hilly (43%), steep (24%)					
Grazing system	continuous (7%), rotational (71%), short grass (22%)					

^1^ two weeks prior sampling; CSHpre, compressed sward height before cutting; CSHpost, compressed sward height after cutting.

**Table 3:** Comparison of observed and predicted herbage mass using original Equations (1) and (2) ^1^, and the newly developed equation based on multiple linear regression (Equation (3)) using dataset 1 (*n* = 1511).

	Observed Herbage Mass	Predicted Herbage Mass			MSPE			CCC		
Equation			RMSE	RMSE	ECT	ER	ED	CCC	ρ	Cb
	(kg DM/ha)	(kg DM/ha)	(kg DM/ha)	(% of Observed Herbage Mass)	(% of MSPE)	(% of MSPE)	(% of MSPE)	From −1 to 1	From −1 to 1	From 0 to 1
1	1218	1223	540	44.3	0.01	24.97	75.03	0.79	0.80	0.99
2	1218	1923	1291	106.0	29.83	53.74	16.43	0.51	0.74	0.69
3	1218	1218	421	34.6	-	-	-	0.83	0.84	0.98

^1^ Equation (1) considers fixed values for herbage DM concentrations (i.e., 16 g/100 g fresh matter) and residual sward height (i.e., 4 cm); Equation (2) considers the measured herbage DM concentration and a fixed residual sward height (i.e., 4 cm). DM, dry matter; RMSE, root mean squared error; MSPE, mean squared prediction error and its partitioning into error due to central tendency (i.e., overall bias; ECT), error due to regression (ER), and error due to disturbance (i.e., random error; ED); CCC, concordance correlation coefficient; ρ, Pearson correlation coefficient; Cb, bias correction factor coefficient.

**Table 4:** Comparison of observed and predicted herbage mass using original Equations (1) and (2) ^1^ using the grouped dataset per paddock (dataset 2; *n* = 360 observations).

	Observed Herbage Mass	Predicted Herbage Mass			MSPE			CCC		
Equation			RMSE	RMSE	ECT	ER	ED	CCC	ρ	Cb
	(kg DM/ha)	(kg DM/ha)	(kg DM/ha)	(% of Observed Herbage Mass)	(% of MSPE)	(% of MSPE)	(% of MSPE)	From −1 to 1	From −1 to 1	From 0 to 1
1	948	1096	452	47.7	10.81	36.51	52.69	0.84	0.88	0.95
2	948	1619	1068	112.7	39.49	49.59	10.92	0.58	0.86	0.68

^1^ Equation (1) considers fixed values for herbage DM concentrations (i.e., 16 g/100 g fresh matter) and residual sward height (i.e., 4 cm); Equation (2) considers the measured herbage DM concentration and a fixed residual sward height (i.e., 4 cm). DM, dry matter; RMSE, root mean squared error; MSPE, mean squared prediction error and its partitioning into error due to central tendency (i.e., overall bias; ECT), error due to regression (ER), and error due to disturbance (i.e., random error; ED); CCC, concordance correlation coefficient; ρ, Pearson correlation coefficient; Cb, bias correction factor coefficient.

**Table 5:** Statistical parameters and coefficient estimate of the newly developed multiple linear regression equation to estimate the herbage mass (kg dry matter/ha) on temperate seminatural, multispecies pastures in Central Europe.

Variable	Unit	Estimate	SE	*p*-Value
Intercept	kg dry matter/ha	−1754	132.0	<0.01
CSH_pre_	mm	17.7	0.52	<0.01
Slope				
flat		0	-	-
hilly		140	62.7	0.03
steep		134	71.1	0.06
Cover	%	15	1.5	<0.01
Altitude	m above sea level	0.39	0.05	<0.01
CSH_pre_ × Slope				
flat		0	-	-
hilly		−3.5	0.66	<0.01
steep		−4.9	0.77	<0.01
Adjusted R^2^		0.71		
RMSE		421 kg dry matter/ha; 35% of observed mean		
MAPE		316 kg dry matter/ha; 35% of observed mean		

CSH_pre_, compressed sward height before cutting; RMSE, root mean squared error; MAPE, mean absolute percentage error; SE, standard error.

## Data Availability

Restrictions apply to the datasets. Data only available upon request due to privacy restrictions.

## Supplementary Materials

The following supporting information can be downloaded at: https://www.mdpi.com/article/10.3390/s24072326/s1, Figure S1. Residuals of herbage mass (relationship between observed and predicted herbage mass) vs. predicted herbage mass on temperate seminatural, multispecies pastures when predicted with the new equation.
