# Supplementary material for: Development of a Robust Sensor Calibration for a Commercially Available Rising Platemeter to Estimate Herbage Mass on Temperate Seminatural Pastures
Source: Sensors (Basel). 2024 Apr 5;24(7):2326. doi: 10.3390/s24072326 (PMC11014092; doi:10.3390/s24072326)
Supplement: Supplementary file 1 [file sensors-24-02326-s001.zip › sensors-2907445-supplementary.pdf]

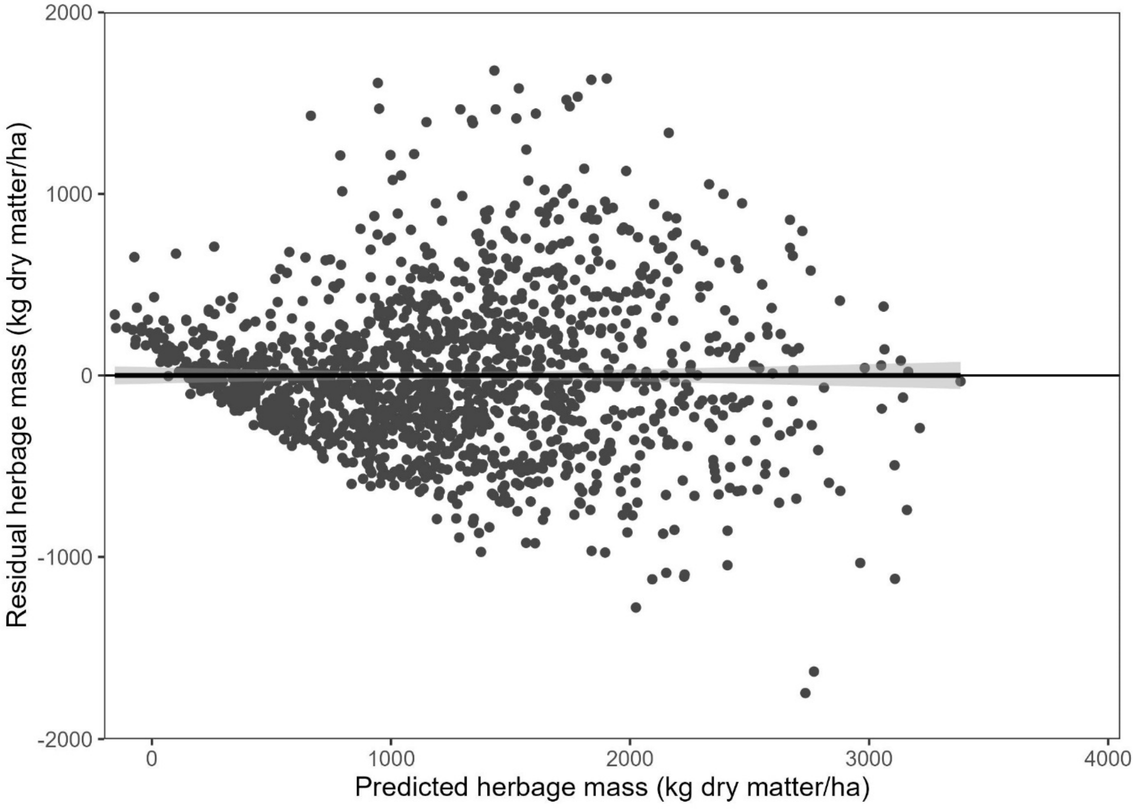

Figure S1. Residuals of herbage mass (relationship between observed and predicted herbage mass) vs. predicted herbage mass on temperate seminatural, multispecies pastures when predicted with the new equation.
